# Gene signatures derived from transcriptomic-causal networks stratified colorectal cancer patients for effective targeted therapy

**DOI:** 10.21203/rs.3.rs-3673588/v1

**Published:** 2023-12-15

**Authors:** Akram Yazdani, Heinz-Josef Lenz, Gianluigi Pillonetto, Raul Mendez-Giraldez, Azam Yazdani, Hanna Sanof, Reza Hadi, Esmat Samiei, Alan P Venook, Mark J Ratain, Naim Rashid, Benjamin G Vincent, Xueping Qu, Yujia Wen, Michael Kosorok, William F Symmans, John Paul Y.C. Shen, Michael S Lee, Scott Kopetz, Andrew B Nixon, Monica M Bertagnolli, Charles M Perou, Federico Innocenti

**Affiliations:** University of Texas Health Science center at Houston

**Keywords:** Patient stratification, gene signature, bevacizumab, cetuximab, colorectal cancer, transcriptomic-causal network, expression quantitative trait loci, Mendelian randomization

## Abstract

Predictive and prognostic gene signatures derived from interconnectivity among genes can tailor clinical care to patients in cancer treatment. We identified gene interconnectivity as the transcriptomic-causal network by integrating germline genotyping and tumor RNA-seq data from 1,165 patients with metastatic colorectal cancer (CRC). The patients were enrolled in a clinical trial with randomized treatment, either cetuximab or bevacizumab in combination with chemotherapy. We linked the network to overall survival (OS) and detected novel biomarkers by controlling for confounding genes. Our data-driven approach discerned sets of genes, each set collectively stratify patients based on OS. Two signatures under the cetuximab treatment were related to wound healing and macrophages. The signature under the bevacizumab treatment was related to cytotoxicity and we replicated its effect on OS using an external cohort. We also showed that the genes influencing OS within the signatures are downregulated in CRC tumor vs. normal tissue using another external cohort. Furthermore, the corresponding proteins encoded by the genes within the signatures interact each other and are functionally related. In conclusion, this study identified a group of genes that collectively stratified patients based on OS and uncovered promising novel prognostic biomarkers for personalized treatment of CRC using transcriptomic causal networks.

## Introduction

Targeted therapies have emerged as a promising therapeutic option in the management of metastatic colorectal cancer (mCRC) when used in combination with chemotherapy [1, 2]. However, the efficacy of targeted therapies can vary significantly among individuals due to their biological heterogeneity [3]. Within the complex landscape of cancer biology, multiple genes and their interconnected pathways collaborate to influence tumor behavior and the response to therapy. incorporating gene interactions into gene expression profiling provides knowledge about cancer biology that can guide clinical care and improve treatment assignments for cancer patients [4–8].

Here, we constructed data-driven networks of gene transcripts and augmented them with germline genotype data. This augmentation, based on the principles of Mendelian randomization, enabled us to build the transcriptomic-causal network [9–12]. The causal network identification facilitates a data-driven approach for discovery of a group of genes that collectively constitute a gene signature for patient stratification. Exploring the network, we identified sub-networks and linked them to the OS to account for effect of confounding genes. We identified gene signatures corresponding to each sub-network, which includes genes impacting OS, and stratified patients under each treatment. We conducted enrichment analysis and explored the biological function of findings using immune signatures known as key inputs in characterizing the immune subtypes of cancer.

We performed this analysis using data from a randomized phase III trial (CALGB/SWOG 80405) comprising 1,165 patients with mCRC treated with cetuximab or bevacizumab combined with chemotherapy (FOLFOX or FOLFIRI). We replicated the findings using data from 103 patients initially excluded from the main analysis along with external cohorts, including the Cancer Genome Atlas (TCGA). We also assessed the biological relevance of interconnectivity among genes using STRING, a protein-protein interaction database. Integrating transcriptomic-causal network in the identification of gene signatures provided significant OS separation among patients. Integrating transcriptomic-causal networks in gene signature identification provided significant OS separation among patients. This approach has improved the biological interpretability and reproducibility of the findings while offering promising prognostic biomarkers to tailor treatment strategies for personalized medicine.

## Results

This study is conducted on a subset of Caucasian samples from CRC patients enrolled in a randomized phase III trial CALGB/SWOG 80405 due to the limited sample size from other ethnicities. Germline genotyped data was extracted from the peripheral blood of 1,165 patients (Figure S1). RNA-seq data were extracted from the primary tumor tissue of 469 patients, obtained from pre-treatment formalin-fixed paraffin-embedded blocks (Figure S2). The trial was designed to compare cetuximab, bevacizumab, or cetuximab + bevacizumab, each plus chemotherapy as first-line therapy in KRAS wild-type advanced or mCRC. The combined arm of the study (cetuximab + bevacizumab) reduced the efficacy of the treatment and therefore, was discontinued prematurely [13]. The clinical study did not show significant differences in OS of patients treated with bevacizumab versus cetuximab. However, these treatments differ at the molecular levels by targeting distinct biological pathways. Therefore, multiple genes and their interconnected pathways collaborate to influence the response to these therapies.

Here, we first constructed a data-driven network of genes and then augmented the network with genetic information to identify the transcriptomic-causal network based on Mendelian randomization technique [9, 11]. Toward this object, we conducted expression quantitative trait loci (eQTL) analysis and utilized the eQTLs as potential instrumental variables to estimate causal relationships among genes and construct the transcriptomic-causal network. We also included known gene regulatory pathways for identifying the causal network. We performed this analysis using the data from patients treated with either bevacizumab or cetuximab. We then assessed the stability of the network and replicated the edges using 103 samples from the bevacizumab + cetuximab arm of the study (Methods). Furthermore, we replicated the interconnectivity among genes using STRING database.

**Figure 1:**
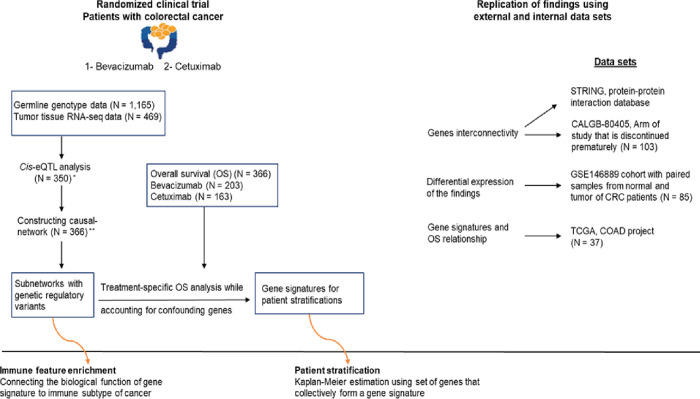
Overall workflow. Gene interactions were revealed as transcriptomic-causal-network and integrated into OS analysis to account for confounding genes and identify gene sets that each collectively impact OS as a gene signature. Gene signatures were used to stratify patients. The results were replicated across multiple replication sets. To elucidate the biological functions of the findings, we explored the association of the identified genes with immune signatures. We also investigated the functional relationships among genes based on the protein-protein interactions database.

By mapping identified eGenes (genes with eQTLs) on the network, we identified sub-networks of genes regulated by eQTLs. We linked the network to OS and estimated the effects of genes on OS that were not attributed to the other genes in the study. Finally, we defined gene signatures corresponding to the sub-networks with genes associated with OS. For the analyses reviewed here, we accounted for the influence of the tumor microenvironment by adjusting for immune cell abundances estimated from RNA-seq data. In addition, all the analyses were adjusted for all RAS and BRAFv600E mutation status along with age, gender. The eQTL analysis is adjusted for batch effect correction. We replicated the findings using a subset of data from the cohort under study as well as two external cohorts. Additionally, we explored the relationship between our findings and immune signatures defined in the literature as key inputs to a description of the immune subtypes of cancer. Figure 1 represents the overall workflow of the study.

### Genetic regulatory variants of gene expression in CRC tumor tissue

**Figure 2:**
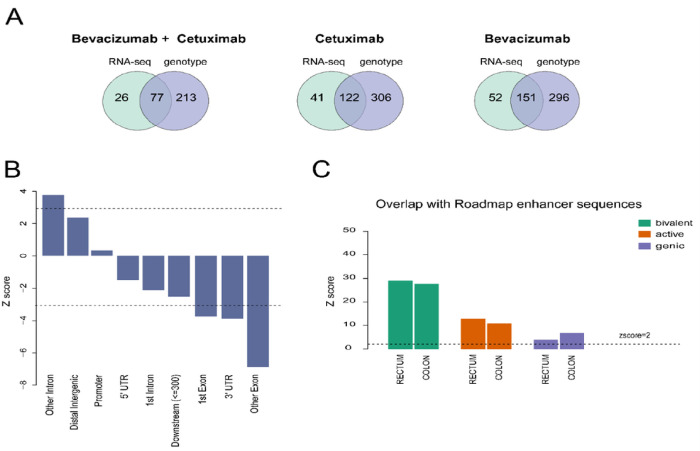
Data and enrichment analysis for cis-eQTL annotation. **A.** The Venn diagrams represent the number of samples with either RNA-seq or germline genotype data in each arm of the study. **B.** Z-score of the enrichment analysis for genomic location. **C.** Z-score of the overlap of cis-eQTLs with Roadmap enhancers for colon and rectum tissue; an active enhancer refers to the regulatory region of DNA that interacts with the promoter DNA region; a genic enhancer refers to regulatory regions in a gene; a bivalent enhancer refers to segments of DNA that have both repressing and activating epigenetic regulators in the same region.

To identify candidate susceptibility genes subject to regulation by genetic variants, we performed a *cis-*eQTL analysis on 8,301 genes with adequate variation (standard deviation of normalized counts across samples > 0.5) while adjusting for covariates. We assessed the relationship of 33,209,829 *cis*-eQTL-gene pairs using 350 Caucasian samples with both RNA-seq and genotype data (Fig. 2A). We selected 352 top genes after applying the permutation test (*p*-value < 0.05) (Figures S4 and S5, Table S1). Enrichment analysis revealed a significant depletion of exons (Fig. 2B), and we observed a high enrichment of cis-eQTLs in bivalent chromatin states associated with enhancer sequences based on the Roadmap Epigenomics Consortium enhancer databases [14] (Fig. 2C). Bivalent enhancer refers to segments of DNA that have both repressing and activating epigenetic regulators in the same region.

### Transcriptomic-causal network

We combined the samples of bevacizumab and cetuximab arms to identify the transcriptomic-causal network since RNA seq data were recorded prior to the treatments (Fig. 2A). We identified the network on 8,301 genes and focused on 2,267 edges that passed the stability assessment. Using the 103 patients (treated with bevacizumab + cetuximab), who were initially excluded from the main analysis, we successfully replicated 71% of the interconnectivity among genes (Methods and Figures S7–9). By taking these steps, we reduced the likelihood of false positive discoveries and increased the chances of reproducibility for the identified edges. We then integrated *cis*-eQTL analysis results for sub-networks identifications and the application of Mendelian randomization technique to identify causal relationships among genes (Methods).

### Gene signatures and patient stratification

**Figure 3:**
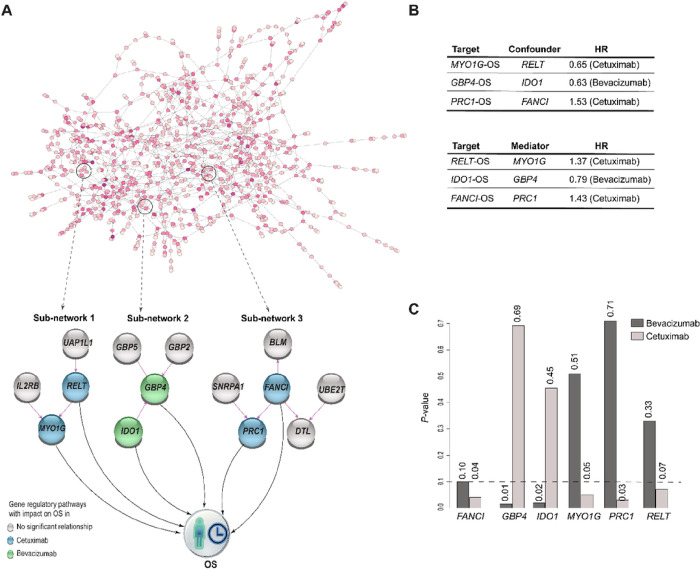
Transcriptomic-causal network linked to OS. **A.** Revealed transcriptomic-causal network and its sub-networks comprising genes with significant effects on OS. **B.** Confounder genes and mediators respect to OS. **C.** Treatment specific p-values for genes with influencing OS within the sub-networks, with significant level represented with dashed line.

We investigated the effect of the gene expression levels on OS by integrating the transcriptomic-causal network with Cox-proportional hazard models for the patients receiving bevacizumab (203 samples) and cetuximab (163 samples) separately. In this way, we controlled for the effects of confounding genes identified using the network. This analysis yielded the identification of three gene signatures corresponding to three sub-networks that comprised genes with significant effects (*p*-value < 0.1) on overall survival (OS) for either bevacizumab or cetuximab treatments (Fig. 3).

One of the sub-networks (Fig. 3A, sub-network 1) includes 4 genes *(UAP1L1, lL2RB, RELT,* and *MYO1G).* Among these genes, *MYO1G* was identified as an eGene. To assess the effect of *MYO1G* on OS, we controlled for the effect of *RELT* as confounding gene and observed that *MYO1G* prolonged, and *RELT* shortened *OS* under cetuximab treatment (HR: 0.65 and 1.37, *p*-value: 0.05, 0.07, respectively) but not under bevacizumab treatment (HR: 0.86 and 1.14, *p*-value: 0.51, 0.33, respectively), (Fig. 3).

The other sub-network (Fig. 3A, sub-network 2) involved 4 genes *(IDO1, GBP2, GBP4,* and *GBP5),* three of which belong to the guanylate-binding protein (GBP) family, including eGene *GBP5.* We observed that the two directly connected genes *IDO1* and *GBP4* significantly prolonged OS of patients under bevacizumab treatment (HR: 0.79, 0.63, *p*-value: 0.018, 0.015, respectively) and not under cetuximab (HR: 0.92, 0.93, *p*-value: 0.455, 0.692, respectively), (Fig. 3C). In this analysis, we controlled for the confounding role of *IDO1* on the *GBP4-OS* relationship.

The third sub-network (Fig. 3A, sub-network 3) includes 6 genes *(BLM, FANCI, UBE2T, SNRPA1, PRC1,* and *DTL),* with *BLM* being an eGene. We observed that *PRC1* and *FANCI* shortened the OS of patients treated with cetuximab (HR: 1.53, 1.43; *p*-value: 0.03, 0.04 respectively) but not with bevacizumab (HR: 0.94, 1.31; *p*-value: 0.71, 0.10), (Fig. 3C).

**Figure 4:**
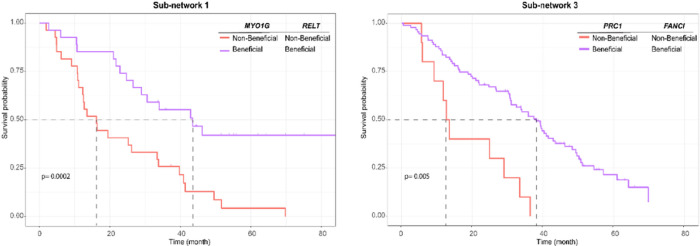
Kaplan-Meier plots based on the identified gene signatures coresponding to each sub-network. The genes are dichotomized into beneficial and non-beneficial based on their third quartile with respect to OS. The purple curve represents the OS for patients with beneficial levels for both genes in the signatures, and the orange curve for those with non-beneficial levels. The median OS exhibited a notable decrease from 43.5 to 16.1 months (*p*-value: 0.0002) in sub-network 1, from 38.1 months to 13.1 months (*p*-value: 0.0059) in sub-network 3, when comparing beneficial to non-beneficial signature level.

To facilitate the nomination of a scoring model for prospective testing as a gene signature and to support the visualization of survival plots, we dichotomized the expression level of each gene based on their third quartile and defined beneficial/non-beneficial expression levels with respect to OS specific to each treatment. We then classified patients based on having either beneficial or non-beneficial expression levels in each sub-network (Methods). We estimated the survival function based on Kaplan-Meier estimator using set of genes that collectively form a gene signature. We observed a notable decrease in the median OS from 43.5 to 16.1 months (*p*-value: 0.0002) for patients with the beneficial vs. non-beneficial levels for both *RELT* and *MYO1G* in sub-network 1; from 38.1 months to 13.1 months (*p*-value: 0.0059) for patients with beneficial vs. non-beneficial levels for both *FANCI* and *PRC1* in sub-network 3 (Fig. 4). For patients with beneficial expression for one gene and non-beneficial expression for other gene in the signatures see Figure S10. Due to the limitations associated with dichotomizing the data in Kaplan-Meier estimators, the statistical power of the analysis for sub-network 2 in the bevacizumab arm was insufficient to detect significant differences among patients.

### Immune feature enrichment

**Figure 5:**
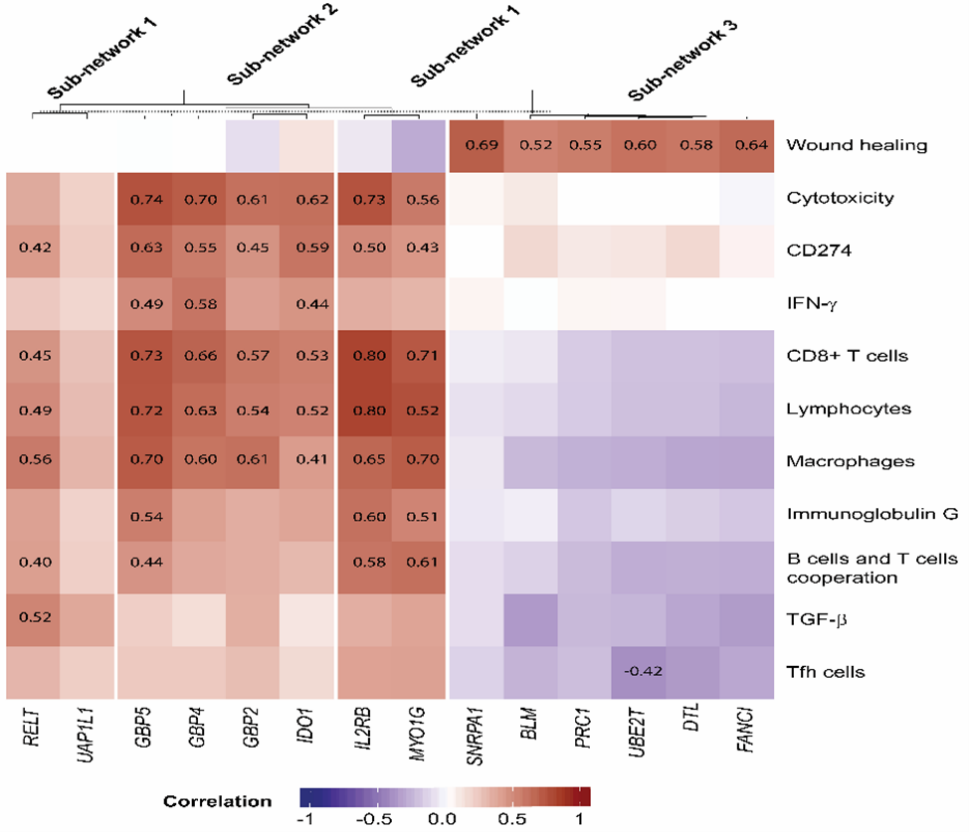
Immune related biological function of genes in the sub-networks 1–3. The heatmap shows the relationship between immune signatures and genes in the sub-networks with impact on OS. The correlations above ± 0.4 are presented on the heatmap.

The biological functions of the gene signatures were assessed by clustering genes in the corresponding sub-networks (Fig. 5) based on 10 immune signatures known as key inputs to a description of the immune subtypes of cancer. These immune signatures including macrophages [15], lymphocytes [16], TGF-β [17], IFN-γ [18], wound healing [18], and CD8 + T cell [19] measure a final common pathway of antitumor immune activity (cytotoxicity [19]), characteriz the immune microenvironment (T follicular helper, Tfh, cells [20]), and mediate the response to checkpoint inhibitors (B cells and T cells cooperation [21]).

The median value of the genes (Tables S2 and S3) within an immune signature was assigned to each patient except for the cytotoxicity signature where the geometric mean was used, according to Rooney *et al.* [22]. In addition, we included immunoglobulin G[23] and single gene CD274[24] as prognosis biomarkers. Since in this study, gene expression levels are adjusted for enriched immune cell types, we first investigated the relationship of the immune signatures and the enriched immune cell types (Figure S11). We then clustered the genes in the sub-networks represented as a heat map plot in Fig. 5.

As the heatmap in this figure shows, Sub-network 1 is divided into two clusters: one includes *IL2RB* and *MYO1G* and the other includes *UAP1L1* and *RELT,* consistent with what we observed in the OS analysis where *MYO1G* prolonged OS and *RELT* shortened OS. Both genes *(MYO1G* and *RELT)* in the corresponding signature, showed stronger correlation with macrophages.

All the gene in sub-network 2 are clustered together and represented strong correlation with cytotoxicity, CD8 + T cells, lymphocytes, and macrophages. The two genes *(IDO1* and *GBP4)* in the corresponding signature showed stronger correlation with cytotoxicity (Fig. 5).

On the other hand, sub-network 3 is only related to wound healing and all the genes in this pathway showed correlations with the wound healing (Fig. 5).

### Replications

In addition to using 103 samples for replication of edges in the network, we used three external replication cohorts. We replicated the edges in the network and investigated the functional relationships among genes within identified signatures using the STRING database, a biological database of protein–protein interactions [25]. Table 1 represents confidence scores for protein interactions corresponding to the genes in the signatures. Scores above 0.5 are indicative of promising evidence for potential physical interactions.

We used the GSE146889 data from the Gene Expression Omnibus database as an external cohort, which includes 85 paired samples from normal and tumor tissue of colorectal cancer patients. For 21,983 genes measured in this cohort, we performed differential expression analysis. All the genes in sub-networks 1–3 except *IL2RB* and *MYO1G* were differentially expressed in this cohort after adjusting for multiple testing using the false discovery rate (FDR) method (FDR < 0.05) (Table 2).

We also replicated the gene-OS relationships using data from the COAD project of The Cancer Genome Atlas (TCGA), consisting of 27 samples treated with bevacizumab and ten with cetuximab. Thus, we performed the replication analysis for the findings related to the bevacizumab arm and not for cetuximab due to the limited number of samples. We used the Wilcoxon rank-sum test to validate the findings for the follow-up time of 27 patients treated with bevacizumab who exhibited elevated expression levels of both *GBP4* and *IDO1* or the absence of high expression levels in both genes. The expression levels were dichotomized based on the third quartile, similar to the Kaplan-Meier analysis. We observed a significant difference between these two groups (*p*-value = 0.038), confirming the association of both *GBP4* and *IDO1* in sub-network 2 with the overall survival of patients treated with bevacizumab.

## Discussion

Prognostic gene expression signatures can help to improve treatment assignments and provide guidance for a personalized treatment-decision. To improve, the interpretability and reproducibility of the gene signatures for patient stratification, we integrated the interconnectivity of genes into analysis by identifying transcriptomic-causal networks. This allowed us to account for the impact of confounding genes on gene-OS relationship and to defined the set of genes that collectively stratifies patients. As a result, we replicated the findings using multiple external cohorts including an external protein-protein interaction database for replication of the functional relationships among genes within gene signatures.

One of the identified gene signatures includes *IDO1* and *GBP4* that are typically expressed at low-to-medium basal levels in the absence of acute activating signals [26]. *IDO1,* with an important role in regulating the innate and adaptive immune response, is overexpressed in many types of cancers, including CRC [27]. Currently, an increasing number of studies have demonstrated that *IDO1* is associated with immune escape by suppressing T cell activity and enhancing regulatory T cells in different tumor types. However, our study revealed that an elevated level of *IDO1* transcription is associated with longer OS in patients treated with bevacizumab. This disparity has been reported in the study of gynecologic and breast cancers [28], which might be due to the activation of tissue-specific gene regulatory pathways in tumor cells. Our study connects the expression of *IDO1* to the expression of 3 genes in the GBP family *(GBP5, GBP4,* and *GBP2)* that all shared the characteristic of high correlation with cytotoxicity signatures. This proposes a new mechanism involved in CRC tumor cytotoxicity, which is not through the IFN-γ pathway related to the negative effect of *IDO1.* That may be potentially activated as a response to bevacizumab therapy.

The other signature includes *MYOIGthat* constitutes the minor histocompatibility antigen HA-2 that binds to MHC class I molecules, makes the antigens recognizable by CD8 + T cells in tumor cells, and allows the destruction of harmful tumor cells [29, 30]. Interestingly, our OS analysis showed positive effects of high expression of *MYO1G* on OS in the cetuximab arm. Its upstream gene in the identified sub-network, *RELT,* is frequently overexpressed in colorectal cancer cell lines and primary colorectal carcinomas [31], consistent with the negative effect of *RELT* on OS shown in our study. *RELT* activates NF-κB pathway and deregulates β-catenin activity in the majority of sporadic forms of colorectal cancer and colon cancer cell lines[31]. β-catenin, on the other hand, has been associated with the expression of MHC class I in glioma stem cells[32]. Given that MHC class I serves as the receptor of HA-2, the interaction between *RELT* and *MYO1G* identified in our study suggests a potential mechanism by which tumor cells can evade immune recognition by CD8 + T cells upon cetuximab therapy.

The protein regulator of cytokinesis 1 *(PRC1)* in the signature corresponding to sub-network 3, reduced OS of patients treated with cetuximab, anti-EGFR therapy. *PRC1* plays an important role in the pathogenesis of various cancers, including colon cancer [33]. *PRC1* and all the genes in its sub-network showed relationships with wound healing signatures, most likely due to their common function in DNA damage and repair. DNA damage sensing and repair dysregulation causes genome instability and is a hallmark of many cancers [34]. In this study, we found that less than 1 *%* of CMS4 tumors carry a beneficial expression level of *PRC1* and *FANCI,* when CMS4 is known for stromal infiltration and resistance to anti-EGFR therapy (Figure S12) [35, 36].

Collectively, our study highlights the significance of data-driven networks in gaining a deeper understanding of the functional mechanisms underlying treatment response. We have identified novel signatures that exhibited a higher potential for reproducibility and improved interpretability. Furthermore, our findings have generated novel hypotheses for experimental testing, shedding light on tumor progression and suppression. Our treatment-specific analysis has also unveiled promising biomarkers for personalized therapies and identified potential targets for the development of new anticancer drugs.

## Materials and methods

### Data

Patients in this study were drawn from the Cancer and Leukemia Group B (CALGB; now a part of the Alliance for Clinical Trials in Oncology) and SWOG 80405 (Alliance) trial. The trial was initiated in September 2005 with a total of 2,326 patients randomized to the three treatment arms (bevacizumab, cetuximab, or their combination in addition to chemotherapy with FOLFIRI or FOLFOX).

### Genotyping.

DNA was extracted from peripheral blood of 2,334 patients. The first genotyping batch was performed on the Illumina HumanOmniExpress-12v1 platform at the Riken Institute (Tokyo, Japan) and included 731,412 genotyped variants. The second genotyping batch was performed on the Illumina HumanOmniExpress-8v1 and included 964,193 SNPs. A total of 719,461 SNPs from HapMap from batch 1 were also on the chip from batch 2. The quality control was performed to remove SNPs with mismatched annotation between the two platforms, genotyping call rates less than 99%, departures from Hardy–Weinberg equilibrium (*P*< 10^−8^), allele frequencies less than 0.05, and individuals with genotyping call rate < 0.90. A total of 540,021 SNPs genotyped for 1,165 samples were remained [37] after passing these filters.

### Tumor RNA sequencing.

Tumor RNA was extracted from pre-treatment formalin-fixed paraffin-embedded (FFPE) tumor blocks (96% primary, 2% metastatic, 2% unknown) from 584 CALGB/SWOG 80405 patients. TruSeq RNA access target enrichment and library preparation protocol were performed using 250 *ng* of template RNA. Sequencing was done using synthesis chemistry targeting 50 M reads with a read length of 2×100 bp per sample on the HiSeq 2500. Data processing was conducted using standard procedures described by Kalari et al.[38].

### Clinical outcomes and covariates.

The primary endpoint of OS was calculated from the time of study entry to death or the last known follow-up for those without reported death. The median follow-up of 640 samples in bevacizumab and cetuximab arms was 65.7 months (95% confidence interval, 63.5–70). In addition, *BRAF* and all *RAS* mutation status were determined by BEAMing (beads, emulsion, amplification, magnetics; Hamburg, Germany) technology [39] and included in the analysis as covariates in addition to age and gender.

## Methods

### Data preprocessing

Among the 584 samples with RNA-seq data, the majority (86%) were Caucasian, with 9% being African American and 5% from other ethnicities. Due to the small sample size of other ethnicities, our analysis focused specifically on primary tumor samples from Caucasian. To ensure data quality and reliability, we excluded genes with low expression variation (standard deviation < 0.5) and low counts across the samples (> 30% zeros). This resulted in a final set of 8301 genes for further analysis. we applied upper quartile normalization, which enabled comparability of gene expression values across different samples. We removed duplicated samples (n = 5) and tumors with low gene expression across the genome (> 50% genes with zero counts; n = 1). Further details and visuals can be found in Figures S1 and S13. We then transformed the RNA-seq data into the log2 scale for the analysis.

To assess the presence of batch effects or hidden population stratification in the RNA-seq data, we conducted principal component analysis (PCA) (Figure S14). In order to validate the self-reported gender information, we utilized k-means clustering based on the expression patterns of genes on chromosome Y. This analysis revealed that 5 samples had a discrepancy between their recorded gender and their biological sex (Figure S15).

Given the influence of the tumor microenvironment on tumor biology, it is crucial to consider the heterogeneity of cell types within the tumor samples when analyzing RNA-seq data. The tumor microenvironment consists of various cell types, including tumor cells, immune cells, stromal cells, and others, which can have distinct gene expression profiles. Therefore, correcting for the abundance of different cell types becomes even more important in order to accurately capture the gene expression patterns specific to tumor cells and to account for any confounding effects introduced by non-tumor cell types. To this end, we estimated the abundance of immune cell types in our RNA-seq data using CIBERSORTx [40] with the validated leukocyte gene signature matrix as a reference. We defined a cell phenotype to be enriched in our data if at most 30% of its estimated scores across samples are zero and its standard deviation is greater than 0.1. As a result, 9 hematopoietic cell phenotypes were enriched in our data: naive and memory B cells, plasma cells, CD8 + T cells, resting and activated memory CD4 + T cells, M0 and M2 macrophages, and activated mast cells [41]. The relationships between the immune cell types and immune signatures are represented in Supplementary Figure S11.

We used 1,165 Caucasian samples with 540,021 SNPs for imputation and employed phased haplotypes from the Haplotype Reference Consortium (HRC) panel through the University of Michigan Imputation Server [42] (https://github.com/statgen/locuszoom-standalone/). Phasing was done using Eagle v2.4 algorithm[43]. The HRC panel combines sequence data across > 32,000 individuals from > 20 medical sequencing studies. The imputed genotype data with imputation score > 0.7 and minor allele frequency (MAF) >0.05 included 5,539,144 common SNPs. These SNPs were used in all the downstream analyses.

For analysis that includes pre-treatment or baseline data (samples with genotype and RNA-seq), we used samples in all arms. However, for the analysis that involved post-treatment data (samples with overall survival and events), we excluded Arm 3 that showed shorter overall survival with two other arms [13] (Figure S16). The comparisons between the population with the RNA-seq and the population without it are presented in Table S4.

### cis -eQTL analysis

To identify germline genetic variants associated with tumor gene expression, we focused on *cis*-eQTL since gene expression is affected by nearby genetic variations [11, 44]. Therefore, for all pairs of genes and SNPs within 1 Mb upstream and downstream of the genes’ transcription start sites (TSS), we applied a linear regression model. In our primary analysis, we estimated latent variables for the potential confounders using the probabilistic estimation of expression residuals (PEER) approach [45]. However, PEER factors did not explain the variation in RNA-seq data in our study (more details in Supplementary PEER Factors section and Figures S17–19).

To address the impact of heterogeneous cell types in our RNA-seq data and to mitigate potential batch effects, we applied several adjustments. We adjusted the expression level of the genes for the enriched cell types in our data estimated by CIBERSORTx [40]. Additionally, we incorporated the first principal component of genotype data to remove the contribution of batch effects that may have arisen during sample processing and sequencing (Figure S3). Furthermore, we considered important covariates such as gender, age, and mutation status (including RAS and BRAFv600E) in our analysis to account for potential confounding factors. This analysis was performed using FastQTL [46]. We applied the adaptive permutation mode of FastQTL while setting for 10,000 permutations and selected eGenes with at least one *cis*-eQTL with an adjusted *p*-value < 0.05 at the gene level. These genes are the ones selected for the OS analysis.

### Enrichment analysis in genomic regions

We investigated the enrichment of identified *cis*-eQTLs in the biological location in DNA, including genic, intron, exon, intergenic, distal intergenic, and upstream and downstream (< = 300 bp) of a gene. To demonstrate that the number of *cis*-eQTLs in any region is higher than expected by chance, we simulated the null distribution by permuting 1,000 random sets of SNPs with the size of *cis*-eQTL matching *cis*-eQTL in terms of allele frequency, gene density, distance from TSS, and density of tagSNPs arising from genomic variability of linkage disequilibrium [47]. We then calculated the Z-score for the observed number of *cis*-eQTL in each region based on the simulated null distribution.

### Overlap of cis-eQTL with enhancer databases

We looked for the overlap of *cis*-eQTLs with enhancer database from the Roadmap Epigenomics Consortium [48]. In particular, we focused on active, genic, and bivalent chromatin states in colon sigmoid, mucosa, and smooth muscle. An active enhancer refers to the regulatory region of DNA that interacts with the promoter DNA region; a genic enhancer refers to regulatory regions in a gene; a bivalent enhancer refers to segments of DNA that have both repressing and activating epigenetic regulators in the same region. We counted the number of top *cis*-eQTLs (the most significant associated SNP per gene) that lie within enhancer sequences in each tissue. We calculated the z-score for each tissue similar to the previous section (Enrichment in the genomic region) and tested the significant levels.

### Transcriptomic-causal networks

The transcriptomic-causal networks are data-driven networks augmented with the principles of Mendelian randomization (MR). The use of transcriptomic-causal networks enables us to uncover the intricate biological connections between genes and identify confounding genes in order to evaluate the direct impact of a gene on overall survival (OS). For the feasibility of constructing robust networks, we initially employed k-mean clustering and clustered genes in 4 classes. We then built a data-driven network for each cluster based on an order-independent implementation of the conditional independence properties, i.e., directed acyclic graph (DAG), learning PC-algorithm [9]. We also augmented the networks with eQTLs as instrumental variables (IVs) to identify causal networks established in the MR principles [11, 49–51]. We identified stable networks by employing two different techniques and then focused on the edges commonly identified by both methods. One method was based on false discovery rate (FDR) where we built a dense network by retaining all the edges within each cluster. We then select significant edges between gene pairs with FDR ≤ 0.05.

The other method for the network stability determination was based on the Hamming distance metric (HD). In this method, we constructed the network for different values of $\alpha_{i}=\left\{10^{-i}|i=3,\ldots ,22\right\}$, and calculated HD for each pair of networks $\left(HD_{ij}\right)$ where j=i−1. Smaller $\alpha_{i}$ leads to a sparser network, but the question is which $\alpha_{i}$ yields the network corresponding to the actual sparsity level. To answer this question, we employed a piecewise regression model by regressing $HD_{ij}$ on $\alpha_{i}$ as follows;

(1) $$HD_{ij}=\beta_{0}+\beta_{1}\alpha_{i}+\beta_{3}\left(\alpha_{i}-\alpha_{k}\right)I_{\left\{\alpha_{k}<\alpha_{i}\right\}}.$$

Here, $I_{\left\{\alpha_{k}<\alpha_{i}\right\}}$ is the indicator function of significant slope change for the breakpoint $\alpha_{k}$. The breakpoint represents a point that indicates a significant change in the slope of the regression model. By fitting this model for all possible breakpoints, $\alpha_{k}s$, we identified the optimal $\alpha_{i}$ corresponds to the maximum breakpoint. Figure 5 represents this procedure for one of the clusters in our analysis, whereas Figure S6 represents the illustration for all the clusters.

### Validating the edges in the network.

To validate the interconnectivity among genes, we used 103 additional patients for the combination arm of the study (bevacizumab + cetuximab plus chemotherapy) excluded from the main analysis. We considered this set as the test set and replicated the interconnectivity among genes using the predictive linear model as follows:

$${\overset{ˆ}{g}}_{\mathrm{test}\;}=G_{test}{\left(G^{\prime }G\right)}^{-1}G^{\prime }g.$$

Here, G is an n×p matrix of the expression level of p predictors for n samples used for building the network. The predictors of each gene (g) refer to its direct upstream and downstream genes in the network. $G_{\mathrm{test}}$ is an m×p matrix of predictors’ expression levels for m samples (here, m=103) selected for the test set. We then calculated the correlation between observed $\left(g_{\mathrm{test}}\right)$ and predicted $\left({\overset{ˆ}{g}}_{\mathrm{test}}\right)$ values, and considered a link validated if the correlation was above 0.5.

### Identification of sub-networks and their regulatory genes.

We defined a sub-network as a set of genes directly connected to an eGene or after one step. We used the eQTLs to implement the Mendelian randomization (MR) technique in addition to the rule of v-structure (details in supplementary V-structure) for identification of causal relationship between genes [11, 49, 50]. The causal relationships discovered genes with a high potential of having a regulatory role in the sub-networks. In addition, we included known gene regulatory pathways for identifying the causal network. For instance, it is known that *FANCI* and *BLM* are in the Fanconi anemia pathway, and *FANCI* regulates *BLM* [52]. Therefore, we used this knowledge to define causal relationship between these two genes in sub-network 3. There were some edges with unidentified directions, but we did not remove those from the study.

### Identifying gene signatures impacting OS.

We performed the Cox proportional hazard model for genes in each sub-network, considering the underlying relationship of the genes in the network by adjusting the analysis for the impact of confounding genes on the gene-OS relationship as

$$h_{l}\left(t\right)=h_{0}\left(t\right)\exp\;\left[\alpha +\;\text{\textbackslash{}varvec}\gamma \tilde{G}+\;\text{\textbackslash{}varvec}\beta G+\;\text{\textbackslash{}varvec}\theta Z+\;\text{\textbackslash{}varvec}\delta T\right],$$

where $\tilde{G}=\left({\tilde{g}}_{1},\ldots ,{\tilde{g}}_{q}\right),\;{\tilde{g}}_{i}=g_{i}\left(I-G_{i}{\left(G_{i}^{T}G_{i}\right)}^{T}G_{i}^{T}\right),\;G=\left(G_{1},\ldots ,G_{q}\right)$. Here, $G_{i}$ includes all the upstream genes of $g_{i}$ in the sub-network, Z represents a matrix of covariates, and T represents a matrix of enriched cell types. This analysis estimated the effect of $g_{i}$ on OS since it included their adjusted expression level, ${\tilde{g}}_{i}$, for the effect of their upstream genes in the sub-network.

In each sub-network, we defined gene signature as the set of genes with significant impacts on OS since these genes collectively impact OS. To facilitate the nomination of a scoring model for prospective testing as a biomarker and to support the visualization of survival plots, we dichotomized the expression level of each gene in each signature based on their third quartile in each arm and defined beneficial/non-beneficial with respect to OS. For instance, if the higher expression of a gene prolonged OS, a patient was stratified as being in a beneficial state if the expression level exceeded the third quartile or as a non-beneficial if it fell below the third quartile. After stratifying patients based on genes in each sub-network, we estimated the overall survival function using the Kaplan-Meier estimator for each treatment.

## Figures and Tables

**Figure 6:**
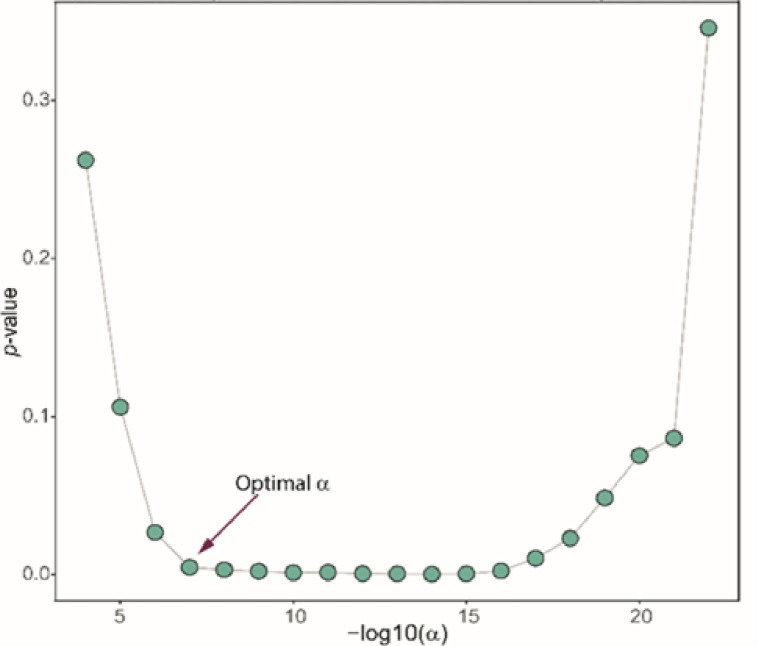
Piecewise regression for assessing stability of the network based on the Hamming distance metric. Y-axis represents *p*-values from model (1) for coefficients *β*_*3*_. X-axis represents −log10 of α_i_.

**Table 1:** Replication of gene interactions in the identified signatures using the STRING database. The confidence scores greater than 0.5 indicate promising evidence for potential physical interactions of proteins encoded by the genes in the signatures.

Protein	Connected	Score
FANCI → PRC1	directly	0.63
IDO1 → GBP4	directly	0.52
RELT → → → MYO1G	within two steps	0.65, 0.51, 0.58

**Table 2:** Summary result of differential expression analysis over 21,983 genes from the GSE146889 database as an external cohort. FC: fold change; SE: standard error; stat: the value of the test statistic for the gene. The adjusted p-value is based on FDR correction.

Gene symbol	log2 (FC)	SE _log2(FC)_	stat	*p*-value	Adjusted *p*-value
**BLM**	−1.75	0.16	−10.98	4.60E-28	9.49E-27
**DTL**	−2.29	0.18	−12.75	3.16E-37	1.47E-35
**FANCI**	−1.88	0.11	−16.75	5.54E-63	4.39E-60
**GBP2**	0.49	0.12	4.27	1.99E-05	5.09E-05
**GBP4**	−0.77	0.15	−5.18	2.25E-07	7.15E-07
**GBP5**	−0.97	0.20	−4.88	1.05E-06	3.11E-06
**IDO1**	−2.35	0.27	−8.62	6.97E-18	6.01E-17
**PRC1**	−2.06	0.13	−15.96	2.44E-57	8.49E-55
**RELT**	−0.47	0.16	−2.90	3.68E-03	6.91E-03
**SNRPA1**	−0.77	0.06	−12.75	3.28E-37	1.52E-35
**UAP1L1**	−0.40	0.11	−3.62	2.91E-04	6.41E-04
**UBE2T**	−2.43	0.16	−15.30	7.35E-53	1.57E-50

## Data Availability

The gene expression data generated in this study are publicly available in Gene Expression Omnibus at GSE196576.
